# Experimental Models for Fungal Keratitis: An Overview of Principles and Protocols

**DOI:** 10.3390/cells9071713

**Published:** 2020-07-16

**Authors:** Micaela L. Montgomery, Kevin K. Fuller

**Affiliations:** 1 Department of Microbiology and Immunology, University of Oklahoma Health Sciences Center, Oklahoma City, OK 73104, USA; Micaela-Montgomery@ouhsc.edu; 2 Department of Ophthalmology, University of Oklahoma Health Sciences Center, Oklahoma City, OK 73104, USA

**Keywords:** fungal keratitis, in vivo models, ex vivo models, in vitro models, *Fusarium*, *Aspergillus*, *Candida*, mouse strain variability

## Abstract

Fungal keratitis is a potentially blinding infection of the cornea that afflicts diverse patient populations worldwide. The development of better treatment options requires a more thorough understanding of both microbial and host determinants of pathology, and a spectrum of experimental models have been developed toward this end. In vivo (animal) models most accurately capture complex pathological outcomes, but protocols may be challenging to implement and vary widely across research groups. In vitro models allow for the molecular dissection of specific host cell–fungal interactions, but they do so without the appropriate environmental/structural context; ex vivo (corneal explant) models provide the benefits of intact corneal tissue, but they do not provide certain pathological features, such as inflammation. In this review, we endeavor to outline the key features of these experimental models as well as describe key technical variations that could impact study design and outcomes.

## 1. Introduction to Fungal Keratitis

Fungal keratitis (FK) is an infection of the cornea and a predominant cause of ocular morbidity and unilateral blindness worldwide [1]. FK is commonplace among agricultural laborers, particularly in tropical climates, who frequently experience corneal abrasions with contaminated vegetative debris. In south India, for example, fungi account for more than half of all corneal ulcers, and the major causative agents are mold species commonly found in the field, particularly *Aspergillus* and *Fusarium* species [2]. The prevalence of the microbial etiology of FK is actually similar in tropical south Florida, but the landscape changes markedly throughout the temperate United States [3]. In these overall cooler regions, the incidence is low (1.2% of microbial ulcers), the major risk factors are contact lens wear (CLW) and ocular surface disease, and the primary causative agent is the commensal yeast *Candida* [4]. Interestingly, however, an outbreak of mold keratitis recently occurred among contact lens wearers throughout the US, Europe, and Singapore, thereby demonstrating that the climactic boundaries for FK are not rigid. In the case of this outbreak, a lens cleaning solution lost its antifungal properties and became contaminated by *Fusarium* sp. commonly found in tap water plumbing systems [5,6]. 

Even when treated promptly, antifungal intervention fails in nearly 50% of all cases, resulting in the need for corneal transplantation or enucleation of the eye if the infection spreads to the intraocular compartments [1,2,3,4,5]. Therefore, the need for better treatment modalities is clear, but their development requires a more complete understanding of host and microbial factors that contribute to disease pathogenesis. In this review, a brief overview of corneal anatomy and FK pathology will lead us into our main discussion concerning experimental systems used to study this devastating eye disease.

### 1.1. Corneal Structure and Function

The cornea is the anterior-most structure of the eye that plays two essential roles in vision. First, it serves as a protective barrier that prevents environmental debris or microbes from damaging intraocular tissues, i.e., the retina. Second, due to its transparent and convex structure, the cornea focuses light onto the retina and accounts for up to 90% of the refractive power in the visual system [7]. 

Anatomically, the cornea is made up of three cellular layers: the outermost epithelium, the central stroma, and the basal endothelium [8]. The corneal epithelium is comprised of 4–5 layers of non-keratinized, simple squamous epithelial cells that are held together by tight intracellular junctions and overlaid by a protective tear film containing mucins (primarily MUC5Ac), lipids, and various antimicrobial compounds [9,10]. Accordingly, a healthy epithelium is highly resistant to microbial invasion and acts as a protective barrier for the deeper corneal layers. The stroma represents 90% of the corneal thickness in humans and is normally transparent due to the careful arrangement of the collagen extracellular matrix (ECM), paucity of resident cells, and avascularity. The sparsely distributed stromal cells include keratocytes that secrete and maintain the ECM, as well various immune cells that respond to microbial invaders [11,12,13,14]. The endothelium serves as a barrier between the stroma and the aqueous humor and facilitates transport between the two; this includes both the diffusion of nutrients into the vessel-free cornea as well as the removal of excess fluid from the stroma in order to maintain its transparency. 

Taken together, the transparency of the cornea is essential for normal vision and is due principally to the unique structural properties of the central stroma. The surrounding epithelial and endothelial layers are in turn critical for maintaining stromal homeostasis [15]. We will next discuss the salient features of FK, which involves a critical breakdown in both corneal barrier and optical functions.

### 1.2. Fungal Keratitis Pathogenesis

FK occurs when fungal spores and/or hyphal fragments bypass the protective epithelium and gain access to the stroma [16]. Thus, epithelial damage is a prerequisite for the development of FK, and this is reflected in the major risk factors already discussed: agriculture-related trauma and contact lens use. Additional risk factors for FK include ocular surface diseases that may alter the barrier function of the epithelium, such as chronic dry eye or Sjogren’s syndrome, as well as topical steroid use and/or systemic immunosuppression [17,18,19]. The pathogenesis of FK is mediated by both the pathogen and host response. Fungal growth is clearly a prerequisite for disease, and that alone may damage the corneal architecture and lead to acute and chronic vision loss. For example, fungal proteases have been detected within the corneas of patients infected with *Aspergillus flavus*, and these proteins are thought to support nutrient assimilation through the direct destruction of the stromal collagen matrix [20,21]. 

The detection of fungal antigens by resident immune cells, namely macrophages and dendritic cells, results in the initiation of a robust innate response, marked primarily by neutrophilic influx that significantly impacts the refractive index of the cornea. Antigen detection is mediated by a variety of pathogen recognition receptors (PRRs), such as Toll-like receptors (TLRs) and C-type lectin receptors (CLRs), which can detect fungal mannan (n-linked), phospholipomannan, α-mannosides, and β-glucans [22,23,24,25]. TLR-4 and CLR Dectin-1 have been shown to play important roles in fungal detection during FK, with TLR-4 driving fungal killing during FK and Dectin-1 signaling resulting in neutrophil and monocyte recruitment to the cornea [24]. Following the resolution of FK, there may be acute or long-term consequences for visual acuity. Long-term visual deficits can be due to corneal scarring, which is mediated by the pro-fibrotic activity of myofibroblasts, as well as the inability of cornea-penetrating blood and lymphatic vessels to resolve after infection, which is documented in fungal, viral, and bacterial keratitis [26,27,28].

In summary, the pathogenesis of FK, similar to any infectious disease, is a complex interaction of the pathogen and host response. Although fungal growth is inherently damaging to corneal tissue, corneal opacification and vision loss is largely a function of the host inflammatory response. In this way, the prevention or rapid resolution of inflammation may represent a critical treatment modality. 

## 2. Experimental Methods Available for FK Research

A critical step in any research design phase is the selection of an appropriate experimental model. In most biological contexts, there are usually two broad system types available: in vivo models, in which a live animal is the study subject, and in vitro models, in which one or more cell type is cultured in isolation. A third approach is available for fungal keratitis research, in which a whole cornea is explanted and studied in a petri dish; this is typically referred to as an ex vivo system. FK research may aim to characterize fungal genes that are important for corneal invasion (i.e., potential antifungal targets), the host response to the fungus, or the utility of a novel treatment modality. The appropriateness of an in vivo, in vitro, or ex vivo approach for these research questions will be discussed, and the details of the models themselves will be described. 

## 3. In Vivo Models

Whereas in vitro and ex vivo systems can provide readouts that are a proxy for disease development, any assessment of the disease itself requires an intact animal. In vivo FK models most completely capture the salient features of FK, including corneal opacification, structural damage, and long-term scarring mediated by host inflammatory processes. While the most common animal model for FK is the inbred mouse, other animals, such as rats and rabbits, have also been used [29,30]. Even within a particular animal system, a myriad of technical variations exist, such as the immune status of the animal or the method of inoculation (Table 1). These variations and their potential impact on the study will be discussed.

### 3.1. Mice 

#### 3.1.1. Mouse Strains

Murine models are the most common in vivo system for FK, which is likely due to the availability of transgenic and knockout lines in combination with their fast reproduction and cost efficiency [29]. Inbred strains of mice, such as BALB/c and C57BL/6, are the most frequently used strains; however, outbred strains such as the NIH Swiss mouse have also been utilized [33]. Several differences exist between these backgrounds that should be considered during the experimental design phase. For example, following corneal injury, Pal-Ghosh et al. demonstrated that reepithelialization in BALB/c mice occurred at a slower rate than in C57BL/6 mice [30]. However, despite this, C57BL/6 mice developed more severe *Aspergillus fumigatus* keratitis than BALB/c mice as indicated by increased inflammatory cell infiltration, increased loss of corneal structure, and an increased expression of markers for macroautophagy, which is a mechanism associated with protein turnover during various stress conditions [31]. This difference in pathological severity between strains is likely attributable to immunological variation. For example, C57BL/6 mice are skewed toward Th1 (T helper type 1) signaling, which is characterized by a strong intracellular immune response. This occurs through the increased expression of cytokines, such as IFNγ (Interferon gamma) and TNFα (Tumor necrosis factor alpha), which are typically protective in the context of bacterial and viral infection. BALB/c mice, on the other hand, are skewed toward Th2 signaling, which is characterized by a robust extracellular immune response. This is mediated by cytokines such as IL(interleukin)-4, IL-5, IL-10, and IL-13, which limit extracellular pathogens, such as parasites and fungi, as well as upregulate antibody production [60,61]. Thus, it appears that in the context of fungal keratitis, Th1 cytokines result in increased inflammation and tissue damage, whereas Th2 cytokines act to decrease inflammation and ultimately reduce the severity of corneal lesions [32,62]. 

Dectin-1 isoforms have been shown to contribute to differences in fungal susceptibility when comparing C57BL/6 mice and BALB/c mice in a model of mucosal candidiasis. For example, Carvalho et al. found that while Dectin-1 ^−/−^ animals were hypersensitive to infection in a C57BL/6 background, Dectin-1 ^−/−^ animals in BALB/c background mice were actually more resistant to infection than wild type [63]. Zhong et al. found that Dectin-1 and TREM-1 (Triggering receptor expressed on myeloid cells 1) acted together to positively regulate Th1 responses in C57BL/6 during FK. The inhibition of this pathway resulted in decreased Th1 cytokines and increased Th2 cytokines, which was associated with a decreased severity of FK in C57BL/6 mice [32]. During FK, Leal et al. revealed that Dectin-1 was not important for fungal killing, but it instead played a role in neutrophil and monocyte recruitment to the cornea in C57BL/6 mice. The recruitment of inflammatory cells during FK contributes significantly to disease pathology, suggesting Dectin-1-signaling as a mechanism for which Th1-skewed C57BL/6 mice experience increased FK severity [24]. 

In a *C. albicans* keratitis study, Wu et al. demonstrated that both outbred NIH swiss mice and BALB/c mice were generally resistant to infection when immunocompetent. Both strains developed infection upon immunosuppression; however, the BALB/c animals displayed a more severe disease course and greater fungal burden than the outbred animals [33]. Due to the prevalence of mouse strains in in vivo models, details relating to the immunosuppression and inoculation techniques below are discussed next.

#### 3.1.2. Inoculation Procedures 

##### Corneal Scratch and Topical Inoculation

**Figure 1:**
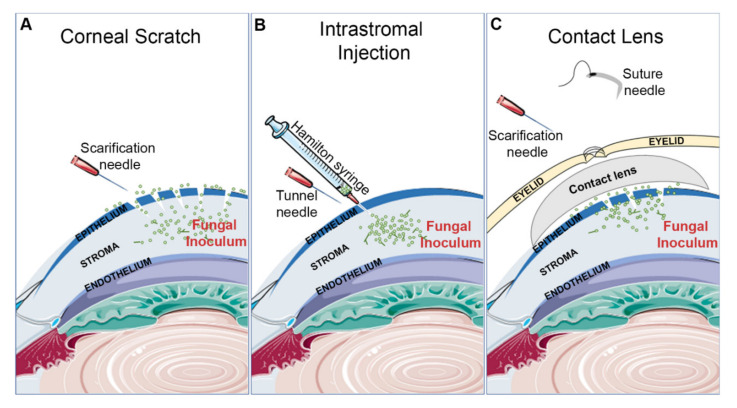
Common inoculation methods for inducing fungal keratitis. (**A**) Corneal scratch: a hypodermic needle is used to abrade the cornea ahead of surface application of the fungal inoculum. (**B**) Intrastromal injection: a hypodermic needle is used to tunnel through the epithelium so that a Hamilton syringe can inject the fungal inoculum directly into the stroma. (**C**) Contact lens: the cornea is scratched, fungal inoculum is added, and the contact lens is placed on the ocular surface. To secure the contact lens, the eyelids are sutured shut.

**Figure 2:**
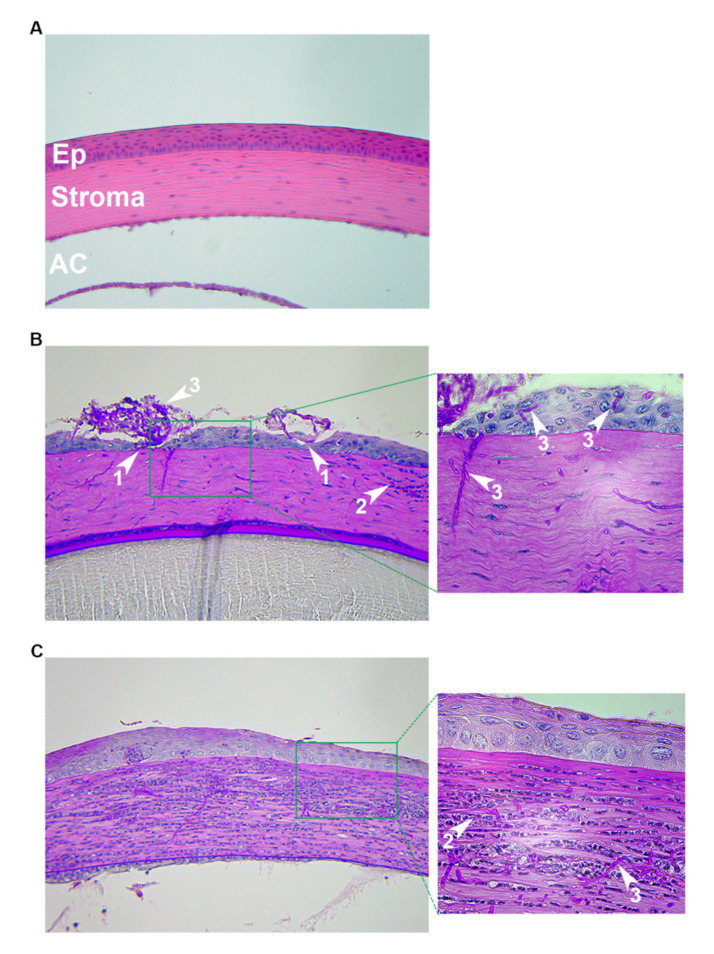
Histopathology following distinct fungal inoculation procedures. 6–8-week-old, immunocompetent, outbred (CD-1, Charles River) mice were used in all panels. (**A**) Uninfected. The normal architecture of the epithelium (Ep), stroma, and anterior chamber (AC) are shown. (**B**) Scratch/surface inoculation: the corneas were first scratched with a needle and 1–2 µL of Aspergillus fumigatus germinated conidia were pipetted onto the ocular surface. Then, a broken toothpick was used to smear the inoculum into the wound. Animals were euthanized, and eyes removed were at 72 h post-inoculation. (**C**) Intrastromal injection: Following a hypodermic needle tunneling through the corneal epithelium, 2 µL (4 × 10^4^ total) of A. fumigatus resting conidia were injected through the tunnel and into the stroma. Animals were euthanized and eyes were removed at 24 h post-inoculation. All images in the left column are 200×; images in the right column are 400× of the designated area. White arrows: 1 = epithelial ulceration; 2 = neutrophil; 3 = fungal hyphae.

Scratching of the cornea with a 26G or 30G needle is the most common mechanism of epithelial debridement for studies concerning *C. albicans* (Figure 1A). However, the extent of abrasion may vary between groups, with some generating 20 or more crosshatch scratches to the central cornea while others reporting just 3, 1-mm deep, scratches [34,35,36]. The inoculum is typically administered as a 5 µl droplet to the surface of the eye, with the inocula ranging between 10^5^ and 10^6^ total yeasts [27,33,34,35,36,37,38,39,40,41]. Scratching has also been used by several groups studying *Fusarium solani* keratitis. In addition to the crosshatching technique mentioned above, a toothpick may be used to further abrade the epithelium and introduce fungal cells into the wound. One such experimental workflow in this regard is as follows: a 2 mm diameter trephine is used to mark the central cornea, and then a size-11 scalpel blade is used to cut into the epithelium within the marked area. Then, a sharpened bamboo toothpick is used to debride and smear *F. solani* mycelium onto the ocular surface [42,43,44,45,46]. Such a method can also be used for *A. fumigatus* as demonstrated in Figure 2B (unpublished data).

The greatest advantage of corneal scratch models is their technical ease of use. Both the abrasions and inoculum can be applied to the ocular surface without the use of a surgical microscope or specialized micropipette system, which contrasts the more technically challenging intrastromal model discussed below. Moreover, corneal abrasions are the major route of infection in humans, thus making the approach clinically relevant. However, a major potential disadvantage of surface inoculation is the inherent inconsistency that may occur across animals. First, even though the scratch procedure and inoculum itself may be well-defined, the number of fungal spores, yeasts, or hyphal fragments that ultimately seed the abrasion is difficult to quantify. Variability in the depth of anesthesia across animals may further impact the duration of inoculum exposure, and hence further amplify inter-animal variability. Second, the surface inoculum (spore suspension or hyphal mat) may contact and infect periocular tissues (e.g., the conjunctiva or eyelids) in some animals, but not others, thereby leading to a qualitatively different disease course. In studies designed to assess the differential pathologies between two groups (e.g., a wild-type versus mutant fungal strain), the large quantitative and qualitative variability between animals of the same group may present a challenge. Increased replicates per group and technical refinement may ultimately overcome such issues. 

##### Intrastromal Injection

Intrastromal injections are used by some groups working with mold species, such as *A. fumigatus*, *A. flavus*, *A. niger*, and *F. solani* (Figure 1B, Figure 2C). The method is generally consistent across the field and includes first using a 30G needle to form a tunnel through the epithelium. Then, a 33G Hamilton syringe is inserted into the tunnel to deliver a 1–2 µL inoculum directly into the underlying stroma. Regardless of fungal species, 10^5^ total conidia is commonly used inoculum, although this can vary by a log-fold either way [1,32,47,48,49,50,51,52]. 

In principle, intrastromal injections can bypass the experimental control issues that might arise with the corneal scratch/surface inoculation model described above (Figure 1 and Figure 2). Not only is a defined number of spores delivered directly into and trapped within the stroma, the inoculum itself should not contact any other ocular tissue. This potentially represents patients who develop FK following a penetrating ocular trauma. However, this approach can be technically demanding due to the precision required to avoid corneal perforation while also administering the appropriate volume of inoculum without air bubbles. A surgical microscope is required and a microinjector system is commonly used to deliver a precise and small volume; the availability of such instruments may present an additional obstacle. 

##### Contact Lens

Contact lens wear is the predominant risk factor for FK in the United States, and several models employing lenses have been developed (Figure 1C). For mice, a lens can be made by taking a 2 mm punch of a commercially available human contact lens. Before placement of the lens, a corneal scratch or intrastromal injection is first used to inoculate the cornea. Most groups using the scratch model debride the cornea at its center; some scraped the epithelium off with a sterile scalpel blade, while others scratched the cornea 3 times with a needle. For the inoculum, 5 µL of a conidial suspension (10^8^/mL for 2 groups using *A. fumigatus*) or a non-specific smear of fungi can be applied to the debrided cornea, followed by contact lens placement. The contact lens must be secured in place by sewing the eyelids shut [31,51,53,54,55]. As an example of one such study, Huang et al. performed an intrastromal injection of 10^8^/mL *F. solani* as described above and then placed a contact lens engineered to have antimicrobial properties onto the eye [56]. 

In the worldwide contact lens-mediated outbreak of *Fusarium* keratitis described above, the infections likely derived from fungal biofilms that formed on the contact lenses while in a plastic storage case [5,6]. Sun et al. recapitulated this scenario by growing a *F. oxysporum* on contact lenses and then placing them on a cornea that had been scratched with a needle [55]. 

Contact lens models are important because of the appreciable risk of developing FK associated with contact lens wear and contact lens solution contamination (Figure 1C). While sewing the eyelids shut is commonly used to hold the contact lenses against the cornea, improved accessibility of 3D printing technology may allow for custom-fitted murine contact lenses to become more prevalent. Variables unique to this model include the effect of eyelid sewing on FK. Furthermore, injury to the eyelids may result in the development of unintended periocular infections. In addition, sewing the eyelid shut may change the temperature and oxygenation of the corneal surface in a way not typically seen with normal contact lens wear. 

#### 3.1.3. Immunosuppression

The use of topical or systemic immunosuppressive drugs is a risk factor for developing FK. Such drugs can similarly be employed in mice to enhance susceptibility to infection in strains that are otherwise resistant [33]. For example, Wu and colleagues rendered BALB/c mice susceptible to *Fusarium solani* keratitis through the administration of neutropenia-inducing drug cyclophosphamide (CP) on days −5, −3, and −1 prior to a corneal scratch-surface inoculation [42]. Animals treated in this way displayed markedly worse clinical scores and increased fungal burden at all tested timepoints post-infection compared to the immunocompetent controls. Interestingly, a similar CP treatment of C57BL/6 mice had the opposite effect of disease outcome in a model of *Aspergillus fumigatus* keratitis. In this case, Leal et al. demonstrated that animals treated with CP at days −3 and −1 pre-infection had increased fungal burden but reduced neutrophilic invasion into the stroma and by extension also had reduced corneal opacity and overt disease [24]. This underscores the contribution of the neutrophilic response in driving disease pathology in animal strains that are inherently susceptible to disease. 

Corticosteroids induce a generalized immune cell defect (e.g., cytokine signaling or phagocytosis) and can also enhance FK sensitivity in mice. For example, Onyewu et al. used 100 mg/kg methylprednisolone on days −5 and −1 before corneal scratch-droplet inoculation with *C. albicans*, and again at day +1, to induce infection in BALB/c animals [38]. The same treatment regimen was similarly used to induce *A. fumigatus* keratitis in BALB/c mice by Rebong et al. [64]. 

#### 3.1.4. Disease Assessment

A key aspect of any in vivo experiment is the assessment of disease severity over time. The combination of clinical, histopathological, microbiological, and cytometric data are all used experimental readouts in FK research models.

*Fungal burden*: The amount of fungus in the cornea may serve as a marker for fungal strain virulence or the capacity of the immune response to control infection. Fungal burden is commonly determined by plating the supernatant from processed enucleated whole eyes or corneas and counting colony-forming units (CFU) on an appropriate growth medium [34,37]. While CFU counting is technically simple and cost efficient, it cannot be assessed more than once in the same animal, i.e., the entire cornea must be removed and homogenized. However, prospective tracking is possible through several approaches, which will be discussed next.

Leal and colleagues generated *A. fumigatus* strains that constitutively express dsRED (red fluorescent protein), the expression of which can be measured and quantified in the cornea with a fluorescence stereomicroscope [24,52]. A green fluorescent protein (GFP)-expressing strain of *Fusarium oxysporum* was used in a similar way [58]. However, in lieu of a reporter strain, a fluorescent probe specific to the fungus may be applied topically and measured by microscopy. For example, Lee et al. conjugated caspofungin, which is a drug that binds the fungal glucan synthase, to the fluorophore 1,3-dichloro-9,9-dimethylacridin-2-one (DDAO) to form a fungal-specific fluorescent probe. At 24 h post-intrastromal injection of *Aspergillus* conidia, animals were anesthetized, and the probe was applied topically to the corneal ulcer. Within 5 min of the application, a fluorescent signal was detectable and quantifiable with an in vivo confocal microscopy; the signal disappeared at 60 min of application [47]. Thus, this method allows for the prospective assessment of fungal burden in the same animal, similar to the fluorescent reporter strain approach. The direct visualization of fungal mycelia through these methods may be a better estimate of fungal burden for filamentous fungi compared to CFU analysis, since colonies that arise on plates may reflect hyphal fragments of variable length. 

*Clinical scoring*: Clinical scoring is a common approach for quantifying disease severity in infected animals. This can be performed using a dissecting microscope or slit lamp to observe disease signs such as size/area of opacity, density of opacity, and surface regularity. Each of these parameters may be considered in assigning a numerical disease score to the animal, and the most frequently used scoring guide is described by Wu et al. [33]. 

*Corneal integrity*: A key feature of FK is the breakdown in the corneal structure as a result of fungal protease activity or inflammation. Second harmonic generation microscopy allowed Zhou et al. to assess the integrity of the cornea during infection. In this technique, a strong signal indicates a healthy arrangement of collagen fibers, while a weak signal indicated a disordered collagen disruption [57]. Abou Shousha et al. used optical coherence tomography (OCT) to quantify corneal thickness (edema) during FK in rats [65]. 

*Inflammation*: Inflammation is a key driver of FK pathology and can be assessed through a variety of techniques. Flow cytometry provides the most direct readout by enumerating various fluorescently immunolabeled cell populations within whole corneas at defined time points [52,57,58]. Various proteins or peptides released by activated leukocytes may serve as a readout for the extent of inflammation. For example, cytokines can be detected at the protein level by ELISA, label-based protein assays, and Western blotting at the RNA level by PCR [32,33,34,36,40,46]. Myeloperoxidase (MPO) is highly abundant in neutrophil granulocytes and other immune cells, and its activity can be measured by commercially available kits; 1 unit of MPO activity corresponds to approximately 2.0 × 10^5^ PMNs [36,40,59]. The release of metalloproteases, which mediate the destruction of the corneal extracellular matrix during infection, can easily be assayed by zymography [43]. 

*Histopathological assessment*: Histology can provide information concerning all of the above-described parameters, including fungal burden, inflammation, and corneal architecture. The most common stain toward this end is Periodic Acid-Schiff (PAS), which stains the carbohydrate-rich cell wall pink or magenta, along with a hematoxylin counterstain (purple) for host cell visualization. Grocott-Gomori Methenamine-Silver (GMS) may also be used to stain fungal cell walls black, and this is typically used in conjunction with hematoxylin and eosin (H&E) staining of serial sections. Immunohistochemistry can more specifically label host and fungal cells through fluorophore-conjugated antibodies [33,34,37,39,40]. 

#### 3.1.5. Antifungal Therapy 

An important proof-of-principal for pathogenesis or translational studies includes the blocking of disease development or treatment of infection once it is established. Toward these ends, experimental antifungal treatments (e.g., drugs or siRNA) can be delivered via a variety of systemic or local routes:

*Intraperitoneal injection*: Intraperitoneal (i.p) injection provides systemic delivery of the drug of interest. For example, to demonstrate the antifungal activity of HDAC inhibitor suberoylanilide hydroxamic acid (SAHA), Li et al. injected mice the compound i.p. at 24 and 48 h following a scratch-and-toothpick inoculation with *F. solani* hyphal fragments (inoculum size not quantitated). The treatment resulted in a decreased disease severity that was associated with a downregulation of TLR-4, TNFα, and IL-1β compared to untreated controls [45].

The major benefits of using i.p. injection is that it is easy to perform and allows for a precise administration of drug that may persist in the animal for a relative long time. Consequently, a single injection of the drug per day, as in the above example, may be adequate to observe an effect. While this degree of technical ease and control is desirable for experimental work, systemic drug delivery may not capture the local/ocular administration routes typically employed in the clinic.

*Topical (droplet) administration*: Eye drops are a common method for delivering antifungal or immunomodulatory drugs (e.g., steroids) in the clinical setting. Thus, the use of droplet administration in vivo may provide an extra level of translatability to the experimental design. For example, tacrolimus is an immunosuppressive drug that acts on T cells and suppresses Th1 cytokines through the inhibition of calcineurin. Two groups have demonstrated that the topical administration of this drug can synergize with standard antifungals in an *A. fumigatus* keratitis model. Zhong et al. demonstrated that a combination therapy of topical voriconazole (10 µg/mL) and topical tacrolimus (0.05% concentration) applied 4 times a day for 7 days decreased corneal inflammation compared to either drug alone [48]. Liang et al. found a similar additive effect using topical tacrolimus (0.05%) twice daily with topical natamycin (5%) 6–8 times daily for up to 21 days [49]. Both studies induced keratitis by intrastromal injection of 2 μL of 1 × 10^5^ *A. fumigatus* conidia. Cyclosporine A (CsA) is another drug that targets calcineurin, and Onyewu et al. demonstrated that the dual treatment of topical treatment of 2% CsA and 0.2% fluconazole (delivered together by droplets 6 times a day) reduced the severity of *Candida* keratitis better than either treatment alone. In this study, infection was initiated in immunosuppressed BALB/c mice by scratching the cornea with a needle and applying 1 × 10^6^ yeasts to the corneal surface [38].

An important consideration for topical administration is that the effective dosage to the ocular surface may be difficult to define due to the rapid clearance or run-off of the droplet. For this reason, droplets are typically administered multiple times a day, as opposed to the once-daily i.p. injections. Thus, the clinical relevance and the technical ease of application of droplets must be weighed against the increased treatment schedule they require. 

*Subconjunctival injection*: Injection of drugs/agents into the conjunctival space may represent a suitable alternative to the topical administration of droplets. Such injections allow for a precise and local delivery of treatment that will not run off the ocular surface. For example, in a study by Taylor et al., neutralizing antibodies to IL-17 or IFNγ were injected subconjunctivally 3 h prior to 2 μL intrastromal injection of 1 × 10^5^ conidia. This study ultimately indicated that IL-17 expression, but not IFNγ expression, was associated with protective immunity following *Aspergillus* keratitis [58]. Zhao et al. found that the subconjunctival injection of fenretinide, a synthetic vitamin A derivative that inhibits the activation of NFKβ’s (Nuclear factor kappa-light-chain-enhancer of activated B cells) proinflammatory signaling cascade, resulted in the protection of corneal transparency during an intrastromal model of *Aspergillus* keratitis utilizing 0.5 × 10^5^ conidia/μL [59]. 

In addition to drugs or compounds, cells can also be delivered to the cornea via sub-conjunctival injection. For example, Zhou and colleagues found that corneal scar resolution following *Fusarium* keratitis was improved by the administration of topical natamycin in combination with subconjunctival injections of umbilical cord mesenchymal stem cells [57]. In this study, infection was induced by scratching the cornea with a needle and toothpick and then applying *F. oxysporum* hyphae to the surface [57].

### 3.2. Rats 

For all of the advantages that mice provide, their corneas are very small and often difficult to manipulate. Larger rodent models have been used for FK studies and may even be the model of choice for some experimental programs. Inoculation techniques such as corneal scratch and intrastromal injection can be used in rats as well as immunosuppression. Some disadvantages of working with rat models are that there are fewer inbred strains available, which in part has resulted in them being less genetically developed than mice. Despite the lack of genetic tools, Wistar rat models can be successfully used for therapeutic testing, assessing host responses during FK, facilitating FK contact lens models, and more [66,67,68,69,70,71]. For example, Zhang et al. established their rat contact lens model by using a scalpel blade to scrape off the cornea epithelium and then depositing *A. fumigatus* mycelia into the wound. Then, a contact lens was adhered to the cornea and the eyelids were sutured shut. Using this model, topical rapamycin-containing liposomes were shown to decrease the severity of corneal lesions during FK [66]. Ahsan et al. developed a new therapeutic consisting of ketoconazole-encapsulated gelatin nanoparticles conjugated to anti-TLR4 and showed that treatment the treatment significantly increased corneal drug retention, decreased inflammation, and increased the resolution of infection [68]. 

### 3.3. Rabbits

Rabbits were initially the go-to animal for ocular research due to their large ocular surface (despite being classified as a small experimental animal), ease of breeding, and ease of handling [72]. Similar to rats, rabbits are also less genetically developed than mice. Inoculation techniques, such as corneal scratch and intrastromal injection, can be used in rabbits as well as various immunosuppressive regimes. Commonly used rabbit strains include pigmented rabbits (*Oryctolagus cuniculus*), New Zealand White rabbits, and Burgundy Fawn rabbits. FK studies using these animals largely focus on diagnostic screening and therapeutic testing [73,74,75,76,77,78,79,80,81,82]. For example, Garcia et al. used rabbits to determine the efficacy of WGA (Wheat germ agglutinin)-peroxidase as a stain for detecting fungal lectins in corneas infected with either *C. albicans*, *A. fumigatus*, or *F. solani* [73]. Ghosh et al. used a rabbit model of FK to determine that the liposomal delivery of Amphotericin B had no increased antifungal activity compared to a standard drug preparation [74].

## 4. Ex Vivo Models

Ex vivo models are particularly useful for addressing questions dealing with the structural features of the cornea. This includes the development of diagnostic imaging, studies that require more biologically and structurally relevant medium for fungal growth and gene expression assays compared to agar plates, and testing antifungals penetration through corneal tissue. Ex vivo experiments can be conducted in whole eyes or corneal/scleral buttons in humans, rabbits, goats, and pigs [83,84,85,86,87], and they can be inoculated with fungi in a manner comparable to in vivo eyes. For example, Blanco et al. infected whole rabbit eyes by an intrastromal injection of 50 µL containing 5 × 10^4^ CFU/mL of *C. albicans* to determine the antifungal properties of a drug cocktail containing tetracycline, chloramphenicol, and colistimethate [87]. Metal rings or artificial anterior chamber systems can be used to stabilize the corneal/scleral button surface. Such an approach has been used to study fungal virulence factors, proteases, and the efficacy of corneal crosslinking as treatment for *Fusarium* and *Candida* keratitis [83,84,85,86,87,88]. 

## 5. In Vitro Models

Live animals consist of multiple cell types whose individual contributions to the pathogenesis of FK may be difficult to resolve. In vitro models bypass this problem through the culture of specific cells of interest, which are isolated from a live animal or human donor within a controlled environment. For example, Taylor and colleagues observed T-cell recruitment within the corneas of *Fusarium* and *Aspergillus*-infected animals and were subsequently interested in the specific cell types responsible for the secretion of various T-cell recruiting chemokines. To address this, they treated fibroblast and macrophage monocultures with IL-17 and found, interestingly, that fibroblasts produce CXCL10 and CCL20, whereas macrophages produce CXCL9 and CXCL10 [58]. Thus, while the in vivo system led to an initial observation concerning FK immunopathology, the in vitro system allowed for the elucidation of specific cellular populations in the process.

In vitro systems are also the model of choice for the dissection of molecular signaling pathways involved in proinflammatory signaling. For example, several groups utilized epithelial cell cultures to demonstrate that those cells become proinflammatory upon interaction with *A. fumigatus* [89,90,91,92,93]. Zhu et al. further showed that treatment with 200 µg/mL of curdlan (soluble β-1,3-glucan) induced IL-6 and TNFα, indicating that the inflammatory response is initiated, in part, through the recognition of this specific fungal cell wall component [90]. Zhao and colleagues used alcohol-inactivated hyphal fragments to demonstrate that the β-1,3-glucan receptor dectin-1 acts synergistically with TLR-2 to induce the inflammatory cascade [92,93]. Li et al. similarly found that LOX-1, a lectin-type receptor similar to CLRs such as dectin-1, was expressed in cornea epithelial cells and treatment with alcohol inactivated hyphal fragments further induced the expression of LOX-1, CXCL1, and TNFα [91].

Following treatment with *C. albicans*, Hua et al. demonstrated that human cornea epithelial cells upregulated reactive oxygen species (ROS) production through an increased activity of p39 MAPK (Mitogen-activated protein kinase) and other oxidative markers [94]. *C. albicans* was used by Qin et al. to identify that IL-17 produced by Th17 cells in vitro activated corneal limbus vascular endothelial cells [41]. 

Stratified human epithelium sheets can also be used to test antifungal drug toxicity. For example, Alshehri et al. found that micafungin and voriconazole had slight drug toxicity in cornea epithelial cells, as indicated by decreased cell viability, wound repair, and increased cell permeability [88].

## 6. Conclusions

The appropriate experimental model for FK research fundamentally depends on the question and desired readout. For example, only in vivo (animal) models include all the cells that reside or invade the cornea during infection and, consequently, they are the only model type available for studying complex disease manifestations (e.g., corneal opacity). As such, in vivo models may be the standard for studies concerning fungal virulence, the dynamics of the inflammatory response, or treatment feasibility. Unfortunately, there is no standardized or ‘universal’ animal model, and those commonly employed vary in ways that can impact the results. The animal species, strain, immune status, inoculation method and size, treatment approach and disease assessment are all parameters that should be considered and perhaps optimized for each study.

For work in which the corneal structure is critical but an intact immune response is not, such as those assessing the ability of drugs to penetrate corneal tissues, ex vivo (explanted) corneas may be sufficient. In vitro models, involving a single host cell type in culture, may be useful for the dissection of specific host pathways involved in fungal recognition and inflammatory signaling. Strides in 3D corneal bioprinting and culturing may further advance in vitro FK models in the near future [95,96]. Overall, the field of FK has a wide array of experimental models that allow researchers to ask and answer complex questions exploring the development, progression, and nuances of this devastating eye disease.

## 7. Experimental Methods Utilized in This Study

The histopathological images depicted in Figure 2 are from previously unpublished experiments. CD-1 outbred animals (6–8 weeks old) were obtained from Charles River laboratories (Wilmington, MA, USA) and housed in the animal facility at Dean McGee Eye Institute with 12h light/dark cycles and access to water and standard chow ad libitum. For both the intrastromal and topical inoculation procedures, animals were anesthetized by i.p injection with ketamine/xylazine. One eye of each animal remained un-inoculated as a control. For the scratch/surface experiment, *A. fumigatus* (strain CEA10) conidia were first germinated by incubating in Yeast extract Peptone (YPD) medium for 3–4 h at 30 °C. The corneas of anesthetized mice were scratched with a 20G needle and 1–2 μL of the inoculum were pipetted onto the ocular surface. A broken, sterile toothpick was then used to smear the inoculum into the wound. The animals were euthanized and the eyes removed at 72 h post-inoculation for PASH (Periodic Acid Schiff and hematoxylin) staining. For the intrastromal inoculation procedure, an 18G hypodermic needle was used to tunnel through the corneal epithelium with the aid of a surgical microscope. 2 μL (4 × 10^4^ total) of *A. fumigatus* (strain CEA10) resting conidia were injected through the tunnel and into the stroma. Animals were euthanized and eyes removed at 24 h post-inoculation and fixed for PASH staining.

All animals were treated in accordance with the guidelines provided in the Association for Research in Vision and Ophthalmology (ARVO) statement for the Use of Animals in Ophthalmic and Vision Research and approved by the Institutional Animal Care and Use Committee (IACUC) at the University of Oklahoma Health Sciences Center (protocol # 18-092-HI; approved 2 June 2019).

## Figures and Tables

**Table 1:** Organization of differing fungal strains, mouse strains, inoculum size, experimental time points, and immunological state used in various fungal keratitis studies.

Fungal Species (Strain ID)	Mouse Strain	Inoculation Procedure	Inoculum Size	Time Points	Immune Status	Cite #
*A. fumigatus* (3.0772)	C57BL/6, BALB/c	Central cornea scraped, inoculum added, soft contact lens added, then eyelids sutured.	5 μL of 1 × 10^8^ CFU/mL	day 1, 3, and 5 p.i.	Immunocompetent	[31]
*A. fumigatus* (3.0772)	C57BL/6	30G needle used to create tunnel into cornea stroma, then 33G Hamilton syringe was inserted into tunnel and inoculum injected.	2 μL of 1 × 10^5^ solution	day 1, 3, and 5 p.i.	Immunocompetent	[32]
*C. albicans* (ATCC 32354)	NIH Swiss, BALB/c	Hypodermic needle used to scarify cornea surface with 30 scratches in a crosshatching grid formation, inoculum applied.	5 μL containing 10^2^, 10^4^, 10^6^, or 10^8^ CFU	6 h; day 1, 4, and 8 p.i.	Immunocompromised with methylprednisolone and cyclophosphamide	[33]
*C. albicans* (SC5314)	C57BL/6	Corneal surface scratched 3 times, 2 mm deep, with a 26G needle, then inoculum added.	5 μL of 1 × 10^5^ CFU solution	6 h; day 1 and 3 p.i.	Immunocompetent	[34]
*C. albicans* (SC5314)	C57BL/6, C57BL/6.129S7-Rag1tm1Mom/J	Corneal surface scratched 3 times with 1 mm incisions using a 26G needle, inoculum added	5 μL containing 1 × 10^5^ CFU	day 1, 3, 5, and 7 p.i.	Immunocompetent	[35]
*C. albicans* (SC5314)	C57BL/6, C57BL/6 TR5 ^−/−^, C57BL/6 Camp ^-/-^	Corneal surface scratched 3 times with 1 mm incisions using a 26G needle, inoculum added	5 μL of 1 × 10^4^ to 10^6^ CFU	6 h; day 1, 3, and 5 p.i.	Immunocompetent	[36]
*C. albicans* (ATCC 32354. SC5314, VE175, Tn7-rim13, BWP17, DAY28)	BALB/c	Hypodermic needle used to scarify cornea surface with 30 scratches in a crosshatching grid formation, inoculum applied.	5 μL containing 1 × 10^5^ or 1 · 10^6^ CFU	6 h, day 1, 4, and 8 p.i.	Immunocompromised with cyclophosphamide	[37]
*C. albicans* (SC5314)	BALB/c, C57BL/6	Hypodermic needle used to scarify cornea surface with 30 scratches in a crosshatching grid formation, inoculum applied.	5 μL containing 1 × 10^6^ CFU	day 1, 3, and 7 p.i.	Immunocompetent	[27]
*C. albicans* (SC5314)	BALB/c	Corneal surface scarified with a 28.5G needle, inoculum applied.	5 μL containing 1 ×10^6^ CFU	day 1, 2, 3, and 4 p.i.	Immunosuppressed with methylprednisolone	[38]
*C. albicans*	C57BL/6	Hypodermic needle used to scarify cornea surface with 30 scratches in a crosshatching grid formation, inoculum applied.	5 μL containing 1 × 10^6^ CFU	day 1 and 5 p.i.	Immunocompetent	[39]
*C. albicans* (SC5314)	C57BL/6	Corneal surface scratched 3 times with 1mm incisions using a 26G needle, inoculum added.	5 μL containing 1 × 10^5^ CFU	day 1, 3, 5, and 7 p.i.	Immunocompetent	[40]
*C. albicans* (MYA-2876)	BALB/c	Hypodermic needle used to scarify cornea surface with 30 scratches in a crosshatching grid formation, inoculum applied.	5 μL containing 1 ×10^6^ CFU	day 1–8 p.i.	Immunocompetent	[41]
*F. solani* (SRL-F2)	BALB/c	Hypodermic needle used to scarify cornea surface with 30 scratches in a crosshatching grid formation, inoculum applied.	5 μL containing 1 × 10^1^, 1 × 10^4^, or 1 × 10^5^ CFU	6 h; days 1, 4, 8, and 14 p.i.	Immunosuppressed with cyclophosphamide	[42]
*F. solani* (SRL-F2)	BALB/c	Hypodermic needle used to scarify cornea surface with 30 scratches in a crosshatching grid formation, inoculum applied.	5 μL containing 1 × 10^5^ CFU	1.5 and 6 h; day 1, 4, and 8 p.i.	Immunosuppressed with cyclophosphamide	[43]
*F. solani* (No.3.1791)	C57BL/6	a 2mm trephine marked the central cornea and a sterile scalpel blade scratched the area. A sharpened bamboo toothpick scraped the area 2-3 times and fungi was then smeared onto the central cornea.	Fungal hyphae ground with a glass rod for fungal suspension, solution adjusted by turbidimeter to get 0.5 Mx suspenion.	day 1, 3, 7, and 10 p.i.	Immunocompetent	[44]
*F. solani* cultures from Henan Eye Institute	C57BL/6	a 2mm trephine marked the central cornea and a sterile scalpel blade scratched the area. A sharpened bamboo toothpick was used to smear fungi onto the central cornea.	Not indicated.	Sacrificed after formation of corneal lesion; varied.	Immunocompetent	[45]
*F. solani*	C57BL/6	a 2mm trephine marked the central cornea and a sterile scalpel blade scratched the area. A sharpened bamboo toothpick scraped the area 2-3 times and fungi was then smeared onto the central cornea.	Not indicated.	6, 12, 18, 24, 36, 72, and 120 h p.i.	Immunocompetent	[46]
*A. fumigatus* (R21), *A. flavus* (DPL9), *A. niger* (DPL29), *F. solani* (DPL114)	SKH1	30G needle used to create tunnel into cornea stroma, then 33G Hamilton syringe was inserted into tunnel and inoculum injected.	2 μL containing 5 × 10^6^ CFU of *A. fumigatus*, *A. flavus*, *A. niger*, or *F. solani*	24 h p.i.	Immunocompetent	[47]
*A. fumigatus* (3.0772)	C57BL/6	30G needle used to create tunnel into cornea stroma, then 33G Hamilton syringe was inserted into tunnel and inoculum injected.	2 μL containing 1 × 10^5^ CFU	8 h; day 1, 3, 5, and 6 p.i.	Immunocompetent	[48]
*A. flavus* and *F. solani*	C57BL/6	30G needle used to abrade the corneal surface, then 33G Hamilton syringe was inserted into the stroma and inoculum injected.	2 μL containing 1 × 10^5^ CFU	48 h p.i.	Immunocompetent	[49]
*A. fumigatus* (3.0772)	C57BL/6	30G needle used to abrade the corneal surface, then 33G Hamilton syringe was inserted into the stroma and inoculum injected.	2 μL of a 5 × 10^4^ conidial/μL solution	12 h; day 1 and 2 p.i.	Immunocompetent	[50]
*A. fumigatus* (3.0772)	C57BL/6	Central cornea scratched with a 25^5/8^G needle and inoculum added. A soft contact lens was applied to the corneal surface and the eyelids were sutured shut.	5 μL of 1 × 10^8^ conidia/μL	12 h; days 1–3, 5, 7, 10, and 14 p.i.	Immunocompetent	[51]
*A. fumigatus* (Af293)	C57BL/6, caspase-1/11 ^−/−^, caspase-11 ^−/−^, IFNAR1 ^−/−^, Dectin-1 ^−/−^, IL-1β ^−/−^, NLRP3 ^−/−^, and ASC ^−/−^	30G needle used to create tunnel into cornea stroma, then 33G Hamilton syringe was inserted into tunnel and inoculum injected.	2 μL containing 1 × 10^5^ CFU	day 1 and 2 p.i.	Immunocompetent	[52]
*A. fumigatus* (3.0772)	C57BL/6	Central cornea was scraped with 30G needle then smeared with fungal colonies. It was then covered with a contact lens and eyelids were sutured shut. Contact lenses were removed after 24 h.	Not indicated.	day 1, 3, and 5 p.i.	Immunocompetent	[53]
*A. fumigatus* (3.0772)	BALB/c	A 2mm scratch was made to the central cornea, covered in inoculum, and contact lens placed. Eyelids were sewn shut.	Not indicated.	day 1 p.i.	Immunocompetent	[54]
*F. oxysporum* (MRL8996)	C57BL/6, IL-1R1 ^−/−^, TLR2 ^−/−^, TLR4 ^−/−^, MYD88 ^−/−^	Lotrafilcon A contact lenses were incubated with conidia, washed, and then incubated further to establish biofilm. The central cornea was abraded and a 2mm punch of the biolfilm contact lens was placed on the central cornea. After 2 h, the contact lenses were removed.	1 × 10^6^ conidia were incubated with the contact lenses.	90 min; 2, 24, and 48 h; day 1, 2, 3, and 4 p.i.	Immunosuppressed with cyclophosphamide	[55]
*F. solani*, *A. fumigatus* (AS 3.772)	C57BL/6	30G needle used to create tunnel into cornea stroma, then 33G Hamilton syringe was inserted into tunnel and inoculum injected. After 12 h, a contact lens fragment was placed on the eye.	2 μL of a 1 × 10^8^ CFU/mL suspension	day 1, 3, and 5 p.i.	Immunocompetent	[56]
*F. oxysporum* (No.3.791)	C57BL/6	a 2mm trephine marked the central cornea and a sterile scalpel blade scratched the area. A sharpened bamboo toothpick scraped the area 2-3 times and fungi was then smeared onto the central cornea.	Not indicated.	day 14, 21, and 28 p.i.	Immunocompetent	[57]
*A. fumigatus* (Af-BP), *F. oxysporum* (8996)	C57BL/6. Rag2 ^−/−^, IL17 ^−/−^	30G needle used to abrade the corneal surface, then 33G Hamilton syringe was inserted into the stroma and inoculum injected.	2 μL containing 1 × 10^5^ conidia	24, 48, and 72 h p.i.	Immunocompetent	[58]
*A. fumigatus* (3.0772)	C57BL/6	Cornea stromas were injected with inoculum.	Volume not specified, 0.5 × 10^5^ conidia/μL	day 1 p.i.	Immunocomptent	[59]
